# Authorship and citation inequities in high-impact emergency medicine journals: a bibliometric analysis

**DOI:** 10.1016/j.afjem.2026.100973

**Published:** 2026-04-22

**Authors:** Sumeyye Cakmak, Ibrahim Sarbay

**Affiliations:** a University of Health Sciences, Istanbul Cam and Sakura City Research and Training Hospital, Department of Emergency Medicine, G-434 Street, 34480, Basaksehir, Istanbul, Turkey; b University of Health Sciences, Istanbul Gaziosmanpasa Research and Training Hospital, Department of Emergency Medicine, Gaziosmanpaşa, Istanbul, 34255, Turkey

**Keywords:** Emergency medicine, Authorship equity, Bibliometrics, Global health, Funding disparities

## Abstract

**Introduction:**

Emergency medicine (EM) is a global discipline; however, marked inequities in authorship representation persist. Disparities between high-income countries (HICs) and lower-and middle-income countries (LMICs) may influence research visibility, access to funding, and scholarly impact. Using recent data, we examined authorship leadership, funding, and citation patterns across national income groups in high-impact EM journals.

**Methods:**

We conducted a cross-sectional bibliometric analysis of articles published between 2020 and 2024 in the 20 highest-ranked EM journals according to Google Scholar Metrics. Bibliographic records were retrieved from PubMed and Web of Science, excluding publication types not considered citable scholarly outputs. Country income classification followed the World Bank 2025 schema based on first-author affiliation. Descriptive statistics and χ² tests assessed distributions across income groups. Multivariable logistic regression identified predictors of LMIC first authorship and funding, while linear regression assessed annual citation counts adjusted for study characteristics.

**Results:**

Among 23,379 eligible articles, first authors were predominantly affiliated with HICs (81.6%), followed by upper-middle-income (10.8%), lower-middle-income (7.3%), and low-income (0.3%) countries. LMIC representation did not increase over time. Larger author teams were inversely associated with LMIC first authorship (*p* < 0.001). In adjusted analyses, LMIC-affiliated first authors accrued fewer annual citations than HIC counterparts (β = −0.79; *p* < 0.001), whereas funded studies were associated with higher citation rates (*p* < 0.001). Africa accounted for 0.6% of publications and did not demonstrate lower citation rates once published.

**Discussion:**

High-impact EM research remains dominated by HIC institutions, with persistent inequities in authorship leadership, funding, and citation visibility. These findings suggest that structural barriers to research leadership and publication may contribute to the observed disparities, rather than differences in scholarly relevance once studies are published. Strengthening LMIC research capacity and promoting equitable collaborations and inclusive publishing policies are essential for a more representative global EM research ecosystem.

## African Relevance

• Africa accounted for only 0.6% (131/23,379) of all emergency medicine publications between 2020 and 2024, making it the least represented WHO region in high-impact EM journals.
• Despite this low publication volume, Africa-focused studies did not demonstrate significantly lower citation rates once published, suggesting that inequities arise primarily at the level of authorship and access to publication rather than scholarly relevance.
• Studies originating from African settings were less likely to receive external funding, reinforcing structural barriers related to research capacity, funding access, and publication resources.
• The observed disparities highlight the need for equitable authorship practices, targeted funding mechanisms, and strengthened research capacity to support African emergency medicine scholarship.
• By explicitly quantifying Africa’s representation within global EM research, this study provides evidence to inform journal policies and global partnerships aimed at improving equity in emergency medicine publishing.

## Introduction

Emergency medicine (EM) is a critical specialty at the intersection of acute care and public health, providing timely diagnosis and treatment for life-threatening conditions across diverse healthcare systems worldwide [1]. As EM has expanded globally, scholarly output has increased; however, inequities in authorship representation persist between high-income countries (HICs) and low-and middle-income countries (LMICs) [1]. Such disparities may affect whose clinical contexts and priorities are reflected in the evidence base, with implications for the global relevance and equity of emergency care scholarship [1,2].

Across medical disciplines, authorship patterns consistently show underrepresentation of LMIC-affiliated authors in research published in high-impact journals. Recent analyses have demonstrated these inequities in cardiology [3], ophthalmology [4], orthopedics [5], and family medicine [6]. Within EM, a bibliometric analysis of global EM publications from 2016 to 2020 similarly documented substantial imbalances in authorship representation, with limited lead authorship from LMIC settings [1].

Multiple structural barriers likely contribute to these patterns, including disparities in research infrastructure, funding, and access to resources for study design, analysis, and dissemination. Open access publishing has been proposed as a mechanism to reduce access barriers and broaden readership [7]. However, open access models frequently involve article processing charges (APCs), which may impose substantial financial barriers for researchers in resource-limited settings and thereby limit equitable participation in publication [8,9].

Despite increasing recognition of these inequities, there remains a need for updated and more granular evaluations of authorship patterns in EM journals using contemporary datasets and broader indicators of research visibility. Prior work in EM has examined authorship representation, and broader scientometric analyses in high-impact medical journals have highlighted persistent diversity gaps in the research ecosystem [2]. Building on this literature, an updated assessment incorporating authorship position, funding, and citation-related indicators across income groups may help identify actionable targets for capacity-building and more equitable research collaboration [1,2]. Therefore, we conducted a cross-sectional bibliometric analysis of publications in top-ranked EM journals from 2020 to 2024 to characterize disparities in authorship, funding, and citation impact across national income levels within high-impact emergency medicine journals.

## Methods

### Study design and data sources

This study was designed as a cross-sectional bibliometric analysis examining authorship, funding, and citation patterns in EM research across national income groups. We identified all publications published between January 1, 2020, and December 31, 2024, in the top 20 EM journals ranked by Google Scholar Metrics under the “Emergency Medicine” category [10].

Bibliographic records were retrieved directly from PubMed and Web of Science using predefined search strategies (Appendix 1). Searches were limited to articles indexed in these databases; Google Scholar was used only for journal ranking and selection, not as a primary data source. Publications were restricted to articles with available bibliographic metadata; no language restrictions were applied at the database-search level. The World Health Organization (WHO) region variable was assigned based on the institutional affiliation of the first author. Studies involving multiple geographic regions (e.g., reviews or multinational analyses) were classified according to the first author’s country to ensure consistency across the dataset. Geographic visualization (Fig. 2) was generated using publicly available map templates and manually curated data, without the use of proprietary or copyrighted mapping software. A full list of the 20 included journals is provided in Appendix 2.

Ethics approval was not required for this study, as all data were obtained from publicly available bibliographic databases and no individual-level or identifiable human data were used.

### Inclusion and exclusion criteria

To capture a comprehensive representation of citable scholarly publications in EM, we excluded publication types that do not typically contribute to indexed scholarly output. We included original research articles, reviews, meta-analyses, case reports, and letters containing scientific or analytical content. Although reviews and meta-analyses are not primary research, they represent substantial scholarly contributions and play a key role in knowledge synthesis and citation dynamics. Letters were included when they contained original data or scientific discussion, as these are relevant to authorship patterns and citation behavior. Case reports and case series were retained as recognized forms of scholarly output in EM and contribute to the dissemination of clinical observations. Excluded categories included editorials, guidelines, consensus statements, interviews, news items, historical articles, corrigenda, retractions, biographies, clinical conference summaries, and authors’ replies.

### Variable definitions

Country income classification was based on the World Bank country income groups as of January 1, 2025, categorized as low-income, lower-middle-income, upper-middle-income, and high-income. Author country affiliation was determined using the institutional affiliation of the first-listed author; when multiple affiliations were reported, the first-listed institution was used for classification.

Publication-level variables included study type (clinical vs. non-clinical), access model (fully open access, hybrid open access, subscription-based), funding status (funded vs. not funded), number of authors, World Health Organization (WHO) region, and assigned Medical Subject Headings (MeSH) terms where available.

Study type and MeSH terms were extracted directly from database records. MeSH terms were available only for publications indexed in PubMed.

### Data extraction and reproducibility

Data extraction was performed using a structured data collection framework. Bibliographic variables were extracted manually from PubMed and Web of Science records by two independent investigators. Screening and data extraction were conducted in parallel, and discrepancies were resolved through consensus. A subset of records was cross-checked to ensure consistency and accuracy of the extracted data. Data consistency was further verified through repeated checks during the data cleaning process. Due to the scale of the dataset, formal inter-rater reliability testing was not performed.

Publications with missing or incomplete author affiliation data were excluded from analyses requiring income classification; the number of excluded records is reported in the Results section.

### Statistical analysis

All statistical analyses were performed using IBM SPSS Statistics 27 (IBM Corp., Armonk, NY, USA). Descriptive statistics were used to summarize publication characteristics, including country income group, authorship position, funding status, access model, study type, number of authors, World Health Organization (WHO) region, and Medical Subject Headings (MeSH) where available. Categorical variables were summarized as frequencies and percentages and compared using chi-square (χ²) tests, with Cramér’s V reported as a measure of effect size.

Multivariable binary logistic regression models were constructed to identify predictors of low- and middle-income country (LMIC) authorship and funding receipt. LMIC authorship was analyzed separately for first and last authorship positions, with each position modeled as a distinct dependent variable. Explanatory variables included study type, access model, funding status, number of authors, WHO region, and publication year. This position-specific modeling approach was chosen to reflect the different roles of first and last authorship in research leadership and senior academic contribution.

Citation impact was assessed using the annual citation rate rather than raw citation counts to reduce bias related to differences in time since publication. The annual citation rate was calculated by dividing the total number of citations received by each publication by the number of years elapsed since its publication. Multivariable linear regression models were then used to examine factors associated with annual citation rates, adjusting for author income group, study type, funding status, number of authors, WHO region, and publication year.

Missing data were handled using a variable-specific complete-case approach. Publications with complete data for the variables required in a given analysis were included in that analysis. Core variables such as income classification, authorship position, funding status, and citation counts were available for nearly all records and were analyzed using the full dataset. Analyses involving variables available only for subsets of publications, such as WHO region or MeSH terms, were restricted to records with available information for those variables. Sample sizes for all analyses are reported in the Results section and corresponding tables and figures. No data imputation was performed, as missingness was primarily related to classification or indexing limitations rather than random measurement error. All statistical tests were two-sided, and a p-value < 0.05 was considered statistically significant.

## Results

A total of 25,209 articles were initially retrieved. After exclusion of non-original publication types (*n* = 1830), 23,379 articles were included in the final analysis (Table 1).

**Table 1:** Descriptive characteristics of the included studies.

Characteristic	n (%)
Publication Type (*N* = 23,379)	
Journal Article	15,338 (65.6)
Letter	3325 (14.2)
Review / Meta-Analysis	2700 (11.5)
Case Report	2016 (8.6)
WHO region of first author affiliation (*N* = 22,832) *	
Americas	10,279 (44.0)
Europe	7053 (30.2)
Western Pacific	2987 (12.8)
South-East Asia	1541 (6.6)
Eastern Mediterranean	841 (3.6)
Africa	131 (0.6)
World Bank classification of first author affiliation (*N* = 23,379)	
High income	19,068 (81.6)
Upper-middle income	2516 (10.8)
Lower-middle income	1714 (7.3)
Low income	81 (0.3)

* Percentages are calculated based on available data. A total of 547 records could not be assigned to a WHO region due to inconsistencies in country naming or limitations in bibliographic database classification. Abbreviation: WHO, World Health Organization.

Most publications originated from high-income settings (Table 1), followed by upper-middle-income (10.8%), lower-middle-income (7.3%), and low-income countries (0.3%) (Table 1). Regionally, most studies were from the Americas (44.0%) and Europe (30.2%), while a smaller proportion originated from other WHO regions, including Africa (0.6%) (Table 1).

### Authorship by country income group

**Fig. 1:**
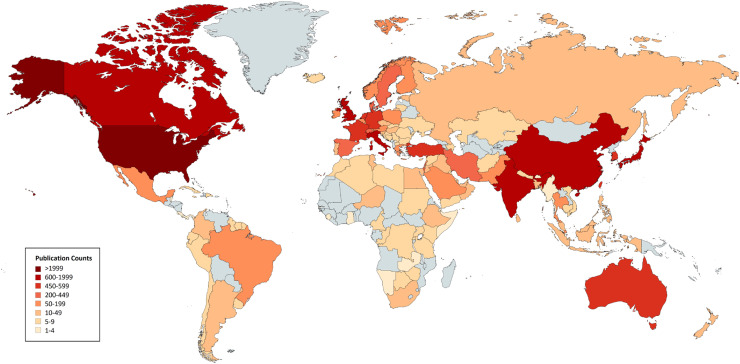
Geographic distribution of first authors in emergency medicine journals (2020–2024). Country-level publication counts are visualized using a log-transformed scale to improve comparability across a highly skewed distribution. Darker shades indicate higher publication output, while countries shown in grey had no first-author representation.

First authors were predominantly affiliated with high-income countries (81.6%), with smaller proportions from upper-middle-income (10.8%), lower-middle-income (7.3%), and low-income countries (0.3%) (Fig. 1). A similar distribution was observed for last authors. The association between author position and income group was statistically significant (χ² = 11.9, *p* = 0.007).

LIC-affiliated first authorship remained low across the study period, ranging from 0.1% to 0.5%, with no statistically significant change over time (*p* = 0.186, Table 2). Publications with larger author numbers were less likely to include first or last authors affiliated with LICs or LMICs (*p* < 0.001, Table 2).

**Table 2:** Study characteristics and proportion of articles with a LIC-affiliated first author.

Characteristic	Articles with a LIC-affiliated first author / All Articles (n)	% with LIC Author	χ²	p-value
Year of publication (*N* = 23,379)			27.7	0.186
2020	4 / 4631	0.1		
2021	19 / 5424	0.4		
2022	24 / 5169	0.5		
2023	13 / 3953	0.3		
2024	21 / 4202	0.5		
Study type (*N* = 23,379)			32.7	0.003
Case Report	2 / 2016	0.1		
Journal Article	63 / 15,338	0.4		
Letter	5 / 3325	0.2		
Review / Meta-analysis	11 / 2700	0.4		
Access model (*N* = 23,379)				
Fully Open Access	39 / 3966	1.0		
Hybrid	40 / 19,156	0.2		
Subscription only	2 / 257	0.8		
Funding status (*N* = 23,379)				
Funded	3 / 2439	0.1		
Not funded	78 / 20,940	0.4		
Number of authors per study (*N* = 23,379)			33.8	<0.001
1	6 / 1136	0.5		
2	5 / 2396	0.2		
3	7 / 3209	0.2		
4	13 / 3081	0.4		
5	14 / 2985	0.5		
6	5 / 2512	0.2		
7	5 / 1906	0.3		
8	7 / 1514	0.5		
9	3 / 1142	0.3		
10	7 / 984	0.7		
11	2 / 628	0.3		
12	1 / 398	0.3		
13	1 / 331	0.3		
14	2 / 224	0.9		
15	1 / 156	0.6		
>15	2 / 777	0.3		

*Abbreviations*: LIC, low-income country; χ², chi-square.

### Predictors of LMIC authorship

In multivariable logistic regression analyses, clinical study type was associated with lower odds of LMIC first authorship (aOR = 0.50, 95% CI 0.44–0.57; *p* < 0.001) and last authorship (aOR = 0.46, 95% CI 0.39–0.52; *p* < 0.001) (Table 2). Increasing author count was also associated with lower odds of LMIC authorship; however, the magnitude of this association should be interpreted with caution due to the sparse representation of LMIC authorship (Table 2).

LIC-affiliated first authorship was observed in 1.0% of fully open-access publications, 0.2% of hybrid publications, and 0.8% of subscription-only publications (Table 2). By funding status, LIC-affiliated first authorship occurred in 0.1% of funded studies and 0.4% of non-funded studies (Table 2).

### Citation discrepancies

In multivariable linear regression models, LMIC affiliation was associated with lower annual citation counts compared with high-income country affiliation (β = −0.79; *p* < 0.001) (Table 3). Citation counts varied by study type, with reviews and meta-analyses associated with higher citation counts (β = 1.41; *p* < 0.001) and letters associated with lower citation counts (β = −0.73; *p* < 0.001) (Table 3).

**Table 3:** Multivariable linear regression analysis of predictors of annual citation counts and funding.

Predictor	Annual Citation Counts		Funding	
	β (95% CI)	p-value	aOR (95% CI)	p-value
World Bank income group (Reference: High-income)				
Low-income country (LIC)	−0.03 (−1.00 to 0.94)	0.952	0.33 (0.08–1.28)	0.107
Lower-middle-income country (LMIC)	−0.79 (−1.17 to −0.41)	<0.001	1.25 (0.72–2.15)	0.429
Upper-middle-income country (UMIC)	0.01 (−0.15 to 0.17)	0.933	1.19 (1.00–1.40)	0.044
Article type (Reference: Clinical study)				
Letter	−0.73 (−0.86 to −0.61)	<0.001	0.32 (0.26–0.38)	<0.001
Review / Meta-analysis	1.41 (1.27 to 1.55)	<0.001	0.76 (0.66–0.87)	<0.001
WHO region (Reference: Americas)				
Africa	−0.15 (−0.92 to 0.62)	0.705	0.68 (0.31–1.48)	0.332
Eastern Mediterranean	0.42 (0.14 to 0.71)	0.003	0.23 (0.16–0.34)	<0.001
Europe	0.43 (0.33 to 0.53)	<0.001	0.40 (0.36–0.44)	<0.001
South-East Asia	0.69 (0.30 to 1.08)	<0.001	0.11 (0.06–0.20)	<0.001
Western Pacific	0.12 (−0.03 to 0.26)	0.114	0.57 (0.49–0.66)	<0.001
Funding support	0.49 (0.35 to 0.64)	<0.001	1.04 (1.04–1.05)	<0.001
Number of authors	0.06 (0.05 to 0.06)	<0.001	1.08 (1.04–1.11)	<0.001
Publication year	−0.42 (−0.45 to −0.39)	<0.001		

High-income countries and the Americas WHO region were used as reference categories in all multivariable models.

Abbreviations: β, beta coefficient; CI, confidence interval; LIC, low-income country; LMIC, lower-middle-income country; UMIC, upper-middle-income country; HIC, high-income country; WHO, World Health Organization.

Regional variation in citation counts was observed. Citation counts for studies from the African region did not differ significantly from the reference region, suggesting that disparities may arise earlier in the research production and publication process rather than reflecting differences in citation performance after publication. Funding support (β = 0.49; *p* < 0.001) and number of authors (β = 0.06; *p* < 0.001) were associated with higher annual citation counts (Table 3).

### Funding patterns

In logistic regression models, upper-middle-income country affiliation was associated with higher odds of funding compared with high-income countries (aOR = 1.19; *p* = 0.044), while no significant differences were observed for low- or lower-middle-income countries (Table 3). Funding odds also varied by study type and WHO region (Table 3).

### MeSH headings

**Fig. 2:**
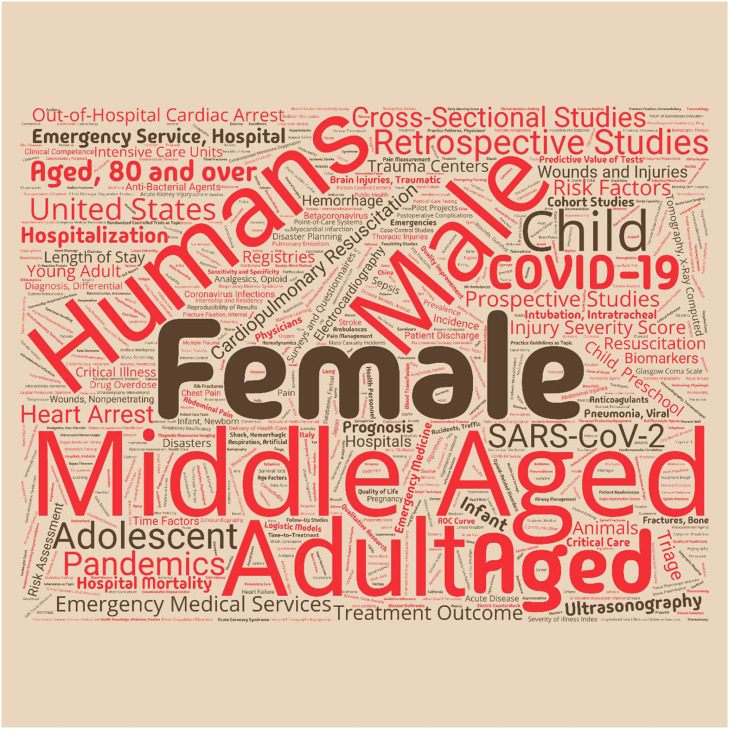
Word cloud of MeSH (Medical Subject Headings) terms from articles with assigned MeSH headings. Word size is proportional to term frequency.

A total of 180,910 MeSH terms were identified across 20,449 publications, with similar distributions across income groups (Fig. 2).

## Discussion

In this bibliometric analysis of high-impact EM journals, we identified persistent and marked inequities in authorship, citation impact, and funding distribution across global income groups. First and last authorship was overwhelmingly concentrated in high-income countries (HICs), while representation from low-income countries (LICs) remained minimal throughout the study period. These disparities were consistent across publication types, access models, and funding status, and were accompanied by lower citation rates and reduced likelihood of funding for publications affiliated with LICs and lower-middle-income countries (LMICs).

Our findings are consistent with prior bibliometric studies in EM and other clinical disciplines, which have reported systematic underrepresentation of LMIC-affiliated authors in high-impact journals despite substantial disease burden in these settings [[1], [2], [3], [4], [5], [6]]. A previous EM-focused bibliometric analysis covering 2016–2020 similarly demonstrated disproportionate leadership by HIC-affiliated authors in global EM research [1]. Comparable patterns have been documented in cardiology, family medicine, ophthalmology, and orthopaedics, suggesting that authorship inequity reflects a broader, cross-disciplinary phenomenon rather than one unique to EM [[3], [4], [5], [6]]. Notably, regional journals such as the African Journal of Emergency Medicine were not included in the Google Scholar Metrics top-ranked list, highlighting potential biases in journal ranking systems that may underrepresent LMIC-focused publications.

When examined at the regional level, African-affiliated research output was markedly lower than that of all other WHO regions, including South-East Asia and the Eastern Mediterranean, underscoring a pronounced imbalance in global EM knowledge production.

Beyond authorship counts, recent work has highlighted that the global EM research agenda itself may be geographically constrained. A mapping review of EM research priority-setting literature reported that most publications originated from North America and Europe and were frequently authored by groups from a single country, despite many claiming a global scope. Importantly, priorities articulated from Africa and Asia tended to focus on a narrower set of region-specific issues, such as infectious diseases and resuscitation, whereas priority agendas in North America and Europe covered a broader range of clinical and nonclinical topics [11]. Together, these findings suggest that both authorship leadership and agenda-setting processes in EM research may insufficiently reflect perspectives from LICs and LMICs. Recent global EM literature suggests that authorship representation is influenced not only by research output but also by structural publication standards, including requirements for local authorship and ethical review [12].

Several structural factors may contribute to the observed disparities. Emergency care research has been framed as a global health priority, particularly for LMICs, given the high burden of time-sensitive conditions and the central role of emergency care systems in achieving population-level health gains and global development targets [13]. However, conducting research in emergency care settings in LMICs entails distinctive methodological, operational, and ethical challenges, including difficulties in defining study populations and outcomes, limitations in data capture and standardization, constrained academic infrastructure, and reduced access to research training and mentorship [13,14]. These challenges may restrict research production and visibility, even in contexts where clinical need is greatest.

Financial barriers related to publishing models may further compound these inequities. Evidence from LMIC researchers indicates that article processing charges (APCs) are widely perceived as unaffordable and may influence journal choice, limit publication opportunities, and affect academic career progression [7,9]. Although open access publishing aims to broaden dissemination, our findings suggest that open access alone does not eliminate authorship inequities, particularly for LIC-affiliated researchers.

Structural limitations within the global research ecosystem also warrant consideration. Emergency departments in LMICs remain underrepresented in most international EM research networks, despite substantial variation in emergency care infrastructure and patient populations across regions. Prior work has proposed that the development of inclusive, emergency department–based global research networks could facilitate the generation of generalizable evidence, support the translation of findings into locally relevant guidelines, nurture future EM researchers, and promote standardization in training and clinical practice [15]. Such networks may represent a pragmatic pathway to improving both the equity and the global relevance of EM scholarship.

The persistent mismatch between disease burden and research output raises ethical concerns regarding equity, representation, and fairness in global knowledge production. Research priorities in emergency care in LMICs have been described as insufficiently defined in the literature, which may contribute to limited research output and visibility from these settings [12,13]. Furthermore, limited inclusion of patients and local stakeholders in research priority-setting processes may reduce the contextual relevance of “global” research agendas [11]. Addressing authorship inequities, therefore, requires not only changes in publishing practices but also broader efforts to strengthen research capacity, ensure fair collaboration, and support locally led scholarship.

## Limitations

This study has several limitations. First, authorship analyses relied primarily on first and last author affiliations, which may underestimate contributions from middle authors and incompletely capture collaborative dynamics. Second, journal selection was based on Google Scholar Metrics, whose ranking methodology lacks transparency and may differ from other bibliometric systems such as Scimago or Journal Citation Reports; this approach may also underrepresent regional or LMIC-based journals [10]. Third, missing affiliation data led to the exclusion of some publications, which may have influenced estimates of representation. Fourth, formal inter-rater reliability assessment was not conducted during data extraction, which may have introduced potential classification bias despite consistency checks. Additionally, data extraction was performed manually, which may be subject to variability despite standardized procedures. The classification of publication types may also introduce conceptual limitations, as categories such as reviews, meta-analyses, and letters differ in their nature and citation dynamics. Furthermore, regression analyses may be affected by model specification choices and the relatively low proportion of LMIC authorship. The extremely low prevalence of LMIC authorship may have resulted in sparse data bias and quasi-complete separation, potentially leading to inflated or unstable odds ratio estimates. These findings should therefore be interpreted with caution, with greater emphasis on the directionality rather than the magnitude of the observed associations. Finally, citation counts were used as a proxy for research impact and may be affected by time since publication, journal visibility, and self-citation practices.

## Conclusions

In conclusion, this study demonstrates persistent inequities in authorship leadership, citation impact, and funding across global income groups within high-impact EM journals. These disparities mirror broader patterns observed across medical disciplines and may reflect structural, financial, and systemic barriers faced by LIC and LMIC researchers. Addressing these gaps will likely require coordinated efforts, including capacity-building initiatives, equitable collaboration models, reduction of financial barriers to publishing, inclusive research networks, and transparent authorship practices. Strengthening locally led EM research is essential to ensure that global EM scholarship reflects the diverse contexts in which emergency care is delivered and contributes meaningfully to improving patient outcomes worldwide.

## Dissemination of results

Findings from this bibliometric study were disseminated through multiple academic and regional channels. Results were presented to members of international emergency medicine research networks and shared with global EM capacity-building initiatives involving African and other LMIC collaborators. A summary of key findings highlighting authorship and funding inequities was circulated among participating institutions and discussed during virtual seminars focusing on equitable publishing practices.

## Funding

This research did not receive any specific grant from funding agencies in the public, commercial, or not-for-profit sectors.

## CRediT authorship contribution statement

**Sumeyye Cakmak:** Writing – original draft, Writing – review & editing, Conceptualization, Methodology, Investigation, Supervision. **Ibrahim Sarbay:** Investigation, Methodology, Writing – review & editing, Data curation, Formal analysis, Visualization, Writing – original draft, Methodology.

## Declaration of competing interest

The authors declare no conflicts of interest.

## Appendix Supplementary materials

Image, application 1
