# Supplementary material for: Authorship and citation inequities in high-impact emergency medicine journals: a bibliometric analysis
Source: Afr J Emerg Med. 2026 Apr 22;16(2):100973. doi: 10.1016/j.afjem.2026.100973 (PMC13123335; doi:10.1016/j.afjem.2026.100973)
Supplement: Supplementary file 1 [file mmc1.docx]

**APPENDIX 1 - Detailed Search Strategies for Literature Identification**

**PubMed Query**

(("Am+J+Emerg+Med"[journal]) OR

("Resuscitation"[journal]) OR

("Ann+Emerg+Med"[journal]) OR

("Disaster+Med+Public+Health+Prep"[journal]) OR

("J+Trauma+Acute+Care+Surg"[journal]) OR

("Intern+Emerg+Med"[journal]) OR

("World+J+Emerg+Surg"[journal]) OR

("West+J+Emerg+Med"[journal]) OR

("Acad+Emerg+Med"[journal]) OR

("J+Intensive+Care"[journal]) OR

("Emerg+Med+J"[journal]) OR

("Eur+J+Trauma+Emerg+Surg"[journal]) OR

("Clin+Toxicol+(Phila)"[journal]) OR

("Scand+J+Trauma+Resusc+Emerg+Med"[journal]) OR

("Curr+Opin+Crit+Care"[journal]) OR

("J+Emerg+Med"[journal]) OR

("Indian+J+Crit+Care+Med"[journal]) OR

("J+Emerg+Nurs"[journal]) OR

("BMC+Emerg+Med"[journal]) OR

("Crit+Care+Clin"[journal]))

AND (("2020/01/01"[Date - Publication] : "2024/12/31"[Date - Publication]))

**Web of Science Query**

SO = (AMERICAN JOURNAL OF EMERGENCY MEDICINE OR RESUSCITATION OR ANNALS OF EMERGENCY MEDICINE OR DISASTER MEDICINE "AND" PUBLIC HEALTH PREPAREDNESS OR JOURNAL OF TRAUMA "AND" ACUTE CARE SURGERY OR INTERNAL "AND" EMERGENCY MEDICINE OR WORLD JOURNAL OF EMERGENCY SURGERY OR WESTERN JOURNAL OF EMERGENCY MEDICINE OR ACADEMIC EMERGENCY MEDICINE OR JOURNAL OF INTENSIVE CARE OR EMERGENCY MEDICINE JOURNAL OR EUROPEAN JOURNAL OF TRAUMA "AND" EMERGENCY SURGERY OR CLINICAL TOXICOLOGY OR SCANDINAVIAN JOURNAL OF TRAUMA RESUSCITATION EMERGENCY MEDICINE OR CURRENT OPINION IN CRITICAL CARE OR JOURNAL OF EMERGENCY MEDICINE OR INDIAN JOURNAL OF CRITICAL CARE MEDICINE OR JOURNAL OF EMERGENCY NURSING OR BMC EMERGENCY MEDICINE OR CRITICAL CARE CLINICS)

PUBLICATION DATE: 2020-01-01 - 2024-12-31

**APPENDIX 2 - List of Emergency Medicine Journals Included in the Study Based on Google Scholar Top Publications**

| **Publication Name** |
| --- |
| The American Journal of Emergency Medicine |
| Resuscitation |
| Annals of Emergency Medicine |
| Disaster Medicine and Public Health Preparedness |
| Journal of Trauma and Acute Care Surgery |
| Internal and Emergency Medicine |
| World Journal of Emergency Surgery |
| Western Journal of Emergency Medicine |
| Academic Emergency Medicine |
| Journal of Intensive Care |
| Emergency Medicine Journal |
| European Journal of Trauma and Emergency Surgery |
| Clinical Toxicology |
| Scandinavian Journal of Trauma, Resuscitation and Emergency Medicine |
| Current Opinion in Critical Care |
| The Journal of Emergency Medicine |
| Indian Journal of Critical Care Medicine |
| Journal of Emergency Nursing |
| BMC Emergency Medicine |
| Critical Care Clinics |
